# Supplementary material for: Chromothripsis during telomere crisis is independent of NHEJ, and consistent with a replicative origin
Source: Genome Res. 2019 May;29(5):737–49. doi: 10.1101/gr.240705.118 (PMC6499312; doi:10.1101/gr.240705.118)
Supplement: Supplemental Material [file supp_gr.240705.118_Supplemental_file_1.zip › contigs/annotated_contigs/DB115/contig.2.DB115_length_633_mean_cov_14.691943128.docx]

**DB115_length_633_mean_cov_14.691943128**

```
TTAAGGGGGTGATTATCTTTCAAATAATCTTTCACATACAATTGTGTATTTATCTGAACATCTACTTATTTATGTTTTTGACCTCAATC
 >chr2:222529940-222530313 - E=9e-213
TAGCCAGGACTACAGATTCTTAAAGTAAATGCACAATAAAAGTCAGTGTTAGTCCTCACTTAGGGTGTGATGAAAGTATAAACAGGTCT

GTTCTCTTTATTTGTGATACCTAAGCCTTTGTTACAGGTTGATATGGTTTGGCTGTGTCCCTGCCCAAATCTCATCTTGAATTCCCACA

TGTTGTGGGAGGGACCCAGTGGGAGGTAATTGAATCATGGGGGCAGGTGTTTCCTGTGCTTTTCTTTTGATAGTGAATAAGCCTCACAA

GATCTAATGGTTT|GATA|GTTATTTTAAAAATTGCTGCTACATCTGCCCTTTACCACTATTTAGGCTTCTCACCAGCTGCCCATGAAT
              >chr2:222529129-222529393 - E=4e-147
TCTCAGTGATAATGAGCTGTGGGTCTCTGAGGGCTGCATTCAATTCCATAAGTGCCCTGTGACATATTATCAGGGCTCATAGAGATGAT

AGCTACATCCAATTTATTTTACGTAATCTCCCTATTTTATGGTGGCTTTTGGTTCTCCAATCACAATTGCTTCTTATTTTAGATCATTT

GTCTAAATGTCA
```
